# Remarks upon the Want of Bony Union in Cases of Fracture of the Neck of the Thigh-Bone within the Capsular Membrane

**Published:** 1826-12

**Authors:** Herbert Mayo


### UNION OF BONES.

*Remarks upon the Want of Bony Union in Cases of Fracture of the Neck of the Thigh-bone within the Capsular Membrane.* By HERBERT MAYO, Esq. &c. &c.

THE body of evidence, adduced in Sir ASTLEY COOPER's valuable work upon Injuries of Joints, sufficiently proves that fractures of the neck of the thigh-bone within the capsular membrane are not followed by bony union : I shall quote a single case in illustration of the general fact.

A woman, about fifty years of age, fell with great violence upon her left hip. The limb was not sensibly shortened, but was rendered useless: pain and swelling followed. She was confined to her bed for the ensuing five months: she then appeared to regain some degree of strength in the injured hip, and gradually became enabled to walk with the assistance of a stick. About thirteen months after the accident, she died suddenly of apoplexy. On examination, the case was found to have been a fracture of the neck of the thigh-bone within the capsular membrane. Union had taken place by means of an intervening layer of soft but tough substance, from two to four lines in thickness; in which, however, nothing like commencing ossification was detected.*

In the preceding case, the long confinement to the recumbent posture would have produced union by bone, had the fracture occurred without the limits of the capsular membrane. What then, we naturally inquire, are the circumstances attending this kind of injury, which essentially modify the usual consequences of fracture, and prevent the bone being knit by the common method?

The points, in which a fracture of the neck of the thigh-bone within the capsular membrane differs from another fracture, appear to consist—

1. In the difficulty of preventing motion between the broken surfaces.

2. In the imperfect supply of blood which the head of the where the means proposed are confined to a single plan of treatment, been unsuccessful. Dr. COLLIN informed me that he had examined ten patients, who had for a long time persevered in performing various exercises, and that they had been scarcely benefited by them. However, there is no doubt that exercises, taken as an *auxiliary* to other plans of practice, are most useful. As to their capability of *curing* a confirmed curvature of the spine, I am every day convinced that the views I have offered on this question, in the Supplement to my work on Distortion, are correct.

* The specimen is in the museum in Great Windmill-street. It was given to me by my friend Mr. Sweatman, who witnessed the whole of the case, and from whom I learnt the details, which I communicated to Sir Astley Cooper.

bone must receive when nourished through the vessels of the ligamentum teres only.

3. In the complete isolation of the fracture, by means of the synovial and capsular membranes; so that the adjacent textures, which are in immediate contact with any other broken bone, can contribute nothing to the reparation of this.

The remarkable case which I have mentioned tends to show that the two first of these peculiarities are insufficient to account for the anomaly under consideration. The ordinary effect of moving a fractured bone is to prevent *every kind of direct union*; yet in this instance union by soft substance had taken place. Soft union, again, having occurred, if it were reasonable to suppose that the insufficient supply of blood to the head of the bone would check the progress of ossification on one side, on the other, at least, we should have expected to find a growth of bony substance. Nothing of this kind, however, existed: on the contrary, the neck of the bone was distinctly shortened.

It remains for us to examine the agency of the adjacent textures in repairing divided parts.

In an earlier Number of the London Medical and Physical Journal, an excellent abstract is given of M. Dupuytren's Observations on the Reunion of Bone, of the exactness of which I entertain no doubt, as they have been confirmed by some important researches of Mr. Brodie's, and correspond accurately with some experiments and dissections of my own.

In simple fracture, the appearances noticed during the first sixty hours are limited to the immediate effects of the injury. An inconsiderable quantity of blood is found effused into the exposed cancelli, and into the neighbouring cellular texture; and the periosteum is seen to be stripped irregularly from the margin of the broken surfaces.

About the fourth day, all the parts surrounding the fracture become thickened and hardened; the cellular substance, the muscular and tendinous fibre, become condensed, so as to form a tough capsule, which contains, and holds in some degree of apposition, the extremities of the broken bone.

The ends of the bone become united by soft substance, which consists in part of the organised clot, if there be any, —in part of lymph effused around the ends of the bone,—in part of a growth from the surface of the investing capsule.

The capsule becomes cartilaginous, and at length ossifies; the muscular and tendinous fibres, which were at first included in it, becoming at the same time distinct and disengaged upon its surface. This change takes place between the third and sixth week.

As yet there is no bony union between the fractured surfaces. The soft substance, by which they are connected, ossifies subsequently, and, as it would appear, through an extension of that process from the bony capsule. The ossification of the intermediate substance takes place between the sixth week and the fifth or sixth month.

In proportion as the intermediate substance becomes ossified, the capsule, or thickening round the united bone, is gradually absorbed.

Thus it appears that certain parts, which are excluded by the synovial membrane from contact with a fractured neck of the thigh-bone, are important agents in repairing other fr actures.

The texture adjacent to a ruptured or divided tendon, or to a divided nerve, is no less instrumental in the union of these parts.

Upon examining, at different periods, the gradual process of restoration which ensues after the division of the tendo Achillis in dogs, the following appearances present themselves. In forty-eight hours, the subcutaneous texture, which contains the divided tendon, is found loaded with coagulable lymph and extravasated blood. On making a longitudinal section of the part, the ends of the tendon are found about an inch asunder, but connected together by clotted blood and swollen cellular membrane. The coagulated blood is in greatest quantity internally, and coheres with the cut surfaces of the tendon. The swollen cellular substance is continuous with the membranous sheath of the tendon above and below the wound.

At the expiration of seven days, the uniting medium remains of greater thickness than the divided tendon, but is readily separable from the skin and from the subjacent muscles. When cut through longitudinally, it appears of a reddish colour, dense, firm, and to a certain degree elastic. It coheres in some parts firmly, in others slightly, with the cut ends of the tendon; but is strongly and inseparably connected with the cellular sheath of the tendon, which is thickened and discoloured for some distance;—so that the sections of the tendon admit, without much force, of being displaced and turned out of their sockets in the uniting membrane, which is left coherent with, as it is obviously produced from, the membranous sheath of the tendon.

After seventeen days, the connecting substance is found to be reduced in thickness: it is firmer and paler than before, and coheres inseparably with either cut surface of the tendon, the glistening fibrous character of which it subsequently assumes.

The appearance of a nerve in process of union, seven days after its division, closely resembles that of a divided tendon.

If a portion, a line in extent, be removed from the infra-orbital nerve of a cat upon the cheek, the wound heals readily; and the sensation of the upper lip, which is temporarily destroyed by the experiment, returns towards the end of the third week. At this period the cut ends of the nervous fibrils appear to be contained in, and united by, a thick mass of tough, grey, semi-transparent substance. On making a longitudinal section of this substance, the ends of the nervous fibrils, which enter it, appear firmly coherent with it, and about one or two lines apart. Here and there a fibril seems to extend further into the connecting medium, but no continuity of nervous substance is as yet re-established between the divided portions.

The portio dura of the seventh nerve, when divided upon the cheek, unites in a similar manner; but it is nearly a month before the appearance of a slow and imperfect action of the orbicularis palpebrarum, upon touching the surface of the eye, shows that the nerve is again becoming capable of transmitting the influence of the will.

The last examples which have been given seem to show, that the adjacent cellular texture is an important agent in repairing divided parts, and, in connexion with what has preceded, make it far from improbable that the exclusion of this substance from contact with the broken neck of the femur is the essential cause of its imperfect restoration. It may serve to confirm this supposition, that, in the remarkable specimen which has been already referred to, *some degree of ossification had taken place externally to the capsular membrane,* which, however, necessarily could not extend to the seat of the fracture: but, on the evidence hitherto brought forward, we can found no more than a plausible conjecture. For inductive proof, it would be requisite to produce a case of deficient union, attended with perfect apposition and absolute rest of the divided parts, with no interruption of the customary supply of blood, and in which the only assignable difference from cases of ready union should consist in the isolation of the divided part from any sort of contact with the cellular texture.

Such a case is not readily met with; but the following instance comes near to include all the conditions required.

A small perforation was made in the cranium of a young cat, immediately before the ear: a thin, but blunted wire, bent at the extremity, was introduced at this aperture, and carried upon the petrous portion of the temporal bone to the side of the pons varolii, so as to divide somewhat more than the anterior half of the fifth nerve in the cranial cavity. The parts supplied by the first and second division of the fifth nerve immediately lost their feeling; and the cornea, in twenty-four hours, became slightly opake. The iris, nevertheless, moved as readily as before the experiment, and the animal evidently retained the vision of the eye, which had lost the sense of touch.

This animal was killed *eighteen months* after the imperfect division of the nerve, during which no additional change had ensued, no increase or diminution of the opacity of the cornea, or return of common feeling.

On opening the cranium, the cut ends of the fibrils of the fifth nerve were found to be not above a line asunder: they were held in this near apposition by the part of the nerve which had not been injured; and they were actually coherent by means of a thin film, which seemed to be a clot of blood that had lost its colouring matter. The cohesion, however, was so slight as to give way to very moderate pressure, and had no resemblance to the firm union described in the preceding instances.

May we not reasonably compare the isolation of a nerve in the arachnoid cavity with the isolation of the neck of the femur in a synovial cavity; and consider the want of efficient union in the instance just detailed, as illustrative and explanatory of that which characterises the important surgical case that has been the theme of so much discussion?

19, *George-street, Hanover-square*; *November 7th*, 1826.

